# Eosinophilic Esophagitis Causing Dysphagia: A Rare Entity in Adults, A Case Report and Review of Literature

**DOI:** 10.14740/gr623w

**Published:** 2014-12-27

**Authors:** Waqas Jehangir, Romel P. Zavala, Abdul I. Mahmad, Mohammed A. Islam, Abdalla Yousif

**Affiliations:** a Raritan Bay Medical Center, Perth Amboy, NJ, USA; b Ross University School of Medicine, Portsmouth, Dominica

**Keywords:** Eosinophilic esophagitis, Dysphagia, Endoscopy

## Abstract

Eosinophilic esophagitis (EE) is an atopic condition of the esophagus that has become increasingly recognized over the last decade. It is associated with a variety of esophageal symptoms such as dysphagia, food impaction and chest pain. Diagnosis of the disorder is dependent on the patient’s clinical presentation and histological findings on esophageal mucosal biopsies. Patients with eosinophilic esophagitis should be referred to an allergist for optimal management since studies suggest a link between allergies and EE. Management includes modifications of diet, medication therapy and mechanical dilation of the esophagus. This brief report provides an overview of the epidemiology, pathophysiology, diagnosis, treatment and a case from our encounter with a patient with EE.

## Introduction

Eosinophilic esophagitis (EE) inflammatory disorder of the esophagus has become increasingly recognized in children and adults over the last decade. This disorder is sometimes referred as primary eosinophilic esophagitis, allergic eosinophilic esophagitis and idiopathic eosinophilic esophagitis. EE is characterized as a dense eosinophilic infiltration in the esophageal mucosa with symptoms of dysphagia, chest pain, epigastric pain and food bolus impaction in adults, while failure to thrive and vomiting is more common in children [1]. Any eosinophils in the esophagus is pathological since eosinophils are absent in healthy individuals. EE is defined as a pathologic disorder characterized by > 15 eosinophils per high power field (HPF) in one or more esophageal biopsy specimens and the absence of pathologic gastrointestinal reflux disease confirmed by a normal pH monitoring study or lack of response to acid-suppression therapy [2]. Eosinophils that infiltrate the esophagus contribute to tissue damage and chronic inflammation. Studies suggest EE shares many clinical and pathophysiologic characteristics with allergies such as asthma [3]. The increasing number of recognized cases of EE has resulted in a dramatic interest and studies to comprehend the etiology of this disorder.

Over the past decade, EE cases were increasingly reported worldwide from the US, Europe, Canada, Australia, Japan and South America. A study on the US population estimated the prevalence of EE to be at 55.0 cases per 100,000 people [4]. Incidence has been increasing for both children and adult but this is debatable. It is unclear if the increase is linked to a genuine phenomenon or increase awareness and recognition of the EE. Moreover, esophageal endoscopic biopsies are currently required to establish the diagnosis of EE and, therefore, variations in endoscopy practices may alter the results of epidemiologic studies. Studies suggest that there is both ethnic and gender variation in the prevalence of EE, with the majority of cases reported in Caucasian males. Patients often have a personal or family history of allergic conditions such as asthma, eczema, atopic dermatitis, seasonal allergies and food allergies [5].

The pathogenesis of EE remains unclear but evidence suggests it is related to the pathogenesis pathway of allergies. EE is a disease associated with T helper cell (Th)-2 type immune response. In particular, elevated levels of the Th-2 cytokines interleukin (IL)-5 with increased levels of eotaxin-3 play a key role in the inflammation process of the esophagus in EE patients [5]. Cytokine IL-5 helps in activation and recruitment of eosinophils into the esophagus. According to Mishra’s animal studies, mice deficient in IL-5 exposed to allergens did not develop eosinophilia in the esophagus. Furthermore, another study, Blanchard showed that the severity of the disease correlated with eotaxin-3 levels. Evidence suggests a genetic susceptibility for the disease since the gene for eotaxin-3, a chemokine involved in promoting eosinophil accumulation and adhesion, showed to be overexpressed in patients with EE compared to controls [2].

EE is also believed to be a mixed immunoglobulin (Ig)E- and non-IgE-mediated allergic response to food and environmental allergens [2]. IgE-mediated reactions are immediate hypersensitivity responses that occur within minutes after exposure to an allergen. On the other hand, non-IgE-mediated allergic disorders have a delayed onset of hours or days after exposure to the antigen and potentially more chronic symptoms. The majority of patients with EE have positive skin prick tests, which detect IgE-mediated reactions, and atopy patch tests, which may identify non-IgE-mediated reactions, to foods and/or aeroallergens [2].

## Case Report

**Figure 1:**
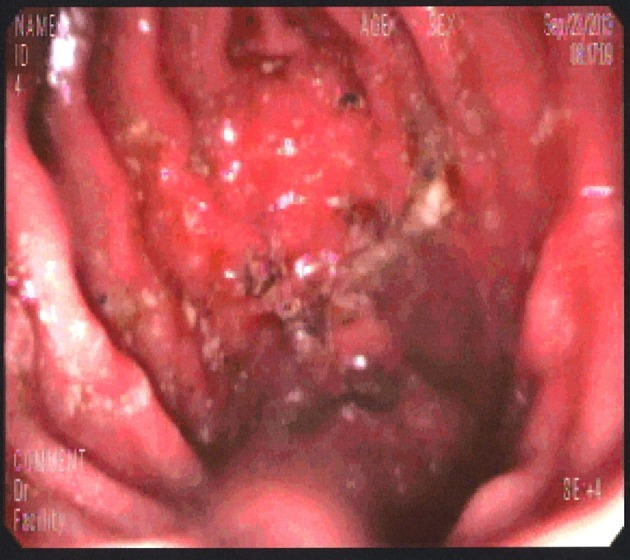
Blood clot just above GE junction with diffuse edema and linear vertical furrows in esophageal mucosa.

**Figure 2:**
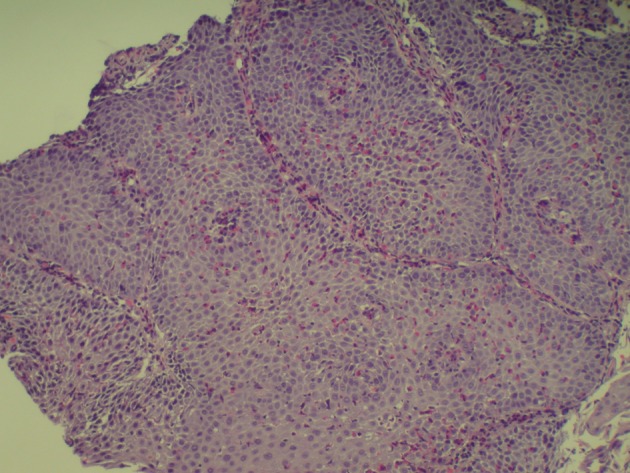
Infiltration of eosinophils features suggestive of eosinophilic esophagitis.

**Figure 3:**
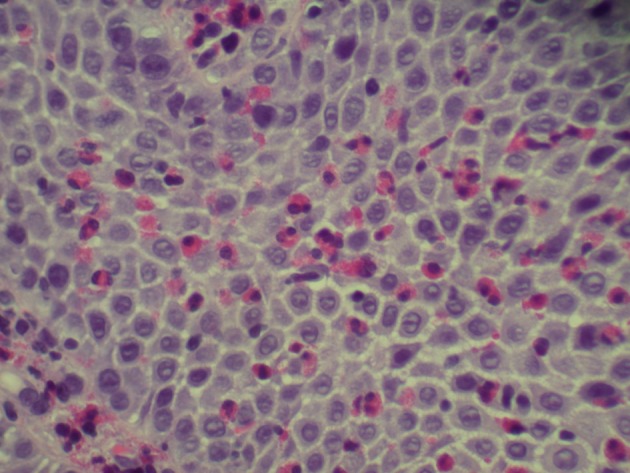
Infiltration of eosinophils (up to 89/HPF) suggesting eosinophilic esophagitis.

A 37-year-old Hispanic male without any significant past medical history presented to emergency room after hematemesis. Before that he had an episode of dysphagia while he was drinking water; at the same time, he felt that the fluid was stuck and he strained to push the fluid down. After a while, he felt a sudden sharp, retrosternal, stabbing chest pain. Soon afterwards he had hematemesis with bright red blood. The patient stated that he had intermittent dysphagia but no hematemesis, hematochezia or melena. Patient denied any change in stool color, nausea, diarrhea or constipation but he had intentional weight loss of almost 100 pounds with diet in the past 4 years. Patient’s mother had lupus but he denied any family history of upper or lower gastrointestinal cancer. Patient was a former cigar smoker but quitted 5 years ago and drinks socially. Physical exam showed blood pressure 136/92 mm Hg, pulse 105/min, respiratory rate 18/min, and temperature 98.2 °F. Patient looked non-toxic but in pain. On abdominal exam, patient’s abdomen was soft with moderate tenderness in the epigastric area but without guarding, rebound tenderness, or hepatosplenomegaly. Complete blood count and basic biochemical test were normal and there was no eosinophilia. Patient was admitted to regular floor and esophagogastroduodenoscopy was performed showing Mallory-Weiss tear, blood clot just above gastroesophageal (GE) junction with diffuse edema and linear vertical furrows in esophageal mucosa (Fig. 1). Biopsies from different levels of esophagus showed fragments of squamous mucosa with marked infiltration of eosinophils (up to 89/HPF) features suggestive of EE (Fig. 2, 3). He was diagnosed with EE with Mallory-Weiss tear and treated with proton-pump inhibitor daily and fluticasone 125 µg inhaler and four puffs swallowed three times a day for 6 weeks. His dysphagia resolved and he remained in remission at the 6-month follow-up.

## Discussion

EE was first reported in 1978 by Landres et al. However, in 1993 Attwood et al recognized EE as a distinct clinical condition [1]. EE is more common in the pediatric population but it is increasingly rising in the adult population in the last decade. Normally there are no eosinophils in the esophagus, therefore their presence indicates pathology. The clinical features in children are broader and can be related to the possibility of children inability to express their symptoms. Children experience nausea, vomiting, poor feeding and regurgitation, while dysphagia (most predominant symptom), food impaction and strictures are more common in the adult population. This can be due to the long-standing inflammation and possible scarring that has gone undetected. Some patients with EE are asymptomatic and suspicion of the disease is based upon incidental findings at endoscopy that is performed for other indications. As a result, diagnosis for EE has been delayed in the past [6].

Many symptoms of EE overlap with GE reflux including eosinophilia in the esophagus. Blanchard found that genes involved in EE were very different from those in gastroesophageal reflux disease (GERD). Patients with EE respond poorly to acid suppression therapy. Since this overlap exists, the diagnosis of EE should be made after GERD has been treated with proton-pump inhibitor or excluded with pH testing. As mentioned before, the majority (about 80%) of patients with EE have a personal or family history of atopic disease and environmental and/or food allergies [2, 7].

Endoscopic findings can help identify patients with EE but it is not a diagnostic tool. Endoscopic features of EE include linear furrowing (which our patient had upon investigation), concentric rings, whitish vesicles scattered over the mucosal surface and exudates, Schatzki ring, small-calibre esophagus, and linear superficial mucosal tears that occur after introduction of the endoscope [7]. Endoscopic mucosal biopsy remains the most important diagnostic test for EE. Two or more biopsies are recommended regardless of the gross appearance of the mucosa, and specimens should be obtained from both the proximal and distal esophagus as well as areas showing endoscopic abnormalities in order to obtain high sensitivity for the detection of EE [6]. As mentioned earlier, diagnosis of EE is characterized by the presence of esophageal such as food impaction, regurgitation, or dysphagia; eosinophilic infiltration of at least 15 eosinophils or more per HPF in the esophageal biopsies of patients who have normal pH studies or are refractory to acid-suppression therapy to rule out GERD [8]. Eosinophils biopsies of patients with GERD have eosinophilic infiltration limited to the distal esophagus with less than 10 per HPF [2] and in our case it was 89/HPF.

The case of EE that was diagnosed in our hospital was based on the endoscopic confirmation of eosinophils infiltration along with symptoms of chest pain and dysphagia. Treatment recommendations include diet restrictions, medications (systemic or topical steroids) and esophageal dilation. Diet restrictions such as avoiding egg, wheat, soy, cow’s milk protein, seafood, peanuts or allergy testing such as skin prick test or atopy patch test have shown promising results in the children population. According to Liacouras studies, 98% of children improved clinically and histologically after the offending allergen had been identified and removed from the diet. Therefore, children and adults with EE can benefit from a complete evaluation by an experience allergist to provide the appropriate therapy. As seen in our case report regarding management, fluticasone is an affective medical treatment for children and adults. In a case series study, all the 21 patients who receive fluticasone two to four puffs twice daily were symptoms free for at least 4 months [2]. Proton inhibitors can also be given as co-therapy for patients who have EE with GERD. A second line of medications are systemic steroids, montelukast, or cromolyn. Endoscopic dilation is a treatment option for those patients with esophageal narrowing that do not respond to medication therapy. Complication of this procedure is mucosal tearing and perforation. In a study, 83% of patients with EE have reported immediate symptomatic improvement after dilation treatment. Patients are periodically followed after treatment due to the nature relapsing symptoms of EE [2].

Therefore, despite recent studies and understanding of EE, the link between allergies and EE still remains unclear. Perhaps, more research in the future regarding IL-5 and exotoxin-3 can lead the way to strengthen the relationship between allergies and EE. EE is rising among the population that it warrants attention to have a clear understanding of its pathophysiology in order to have pure recognition of the disease and open new doors to create effective treatments and management for patients.
